# Inequities in COVID-19 Vaccination Rates in the 9 Largest US Cities

**DOI:** 10.1001/jamahealthforum.2021.2415

**Published:** 2021-09-03

**Authors:** Adam Sacarny, Jamie R. Daw

**Affiliations:** 1 Department of Health Policy and Management, Columbia University Mailman School of Public Health, New York, New York; 2 National Bureau of Economic Research, Cambridge, Massachusetts

## Abstract

This cross-sectional study evaluates neighborhood-level data in the 9 largest US cities to identify inequities in COVID-19 vaccination rates.

## Introduction

The equitable receipt of COVID-19 vaccinations is a national priority.^1^ Most jurisdictions in the United States report limited data on vaccinated people, impeding assessment of vaccination equity.^2^ We used neighborhood-level data to estimate inequities in COVID-19 vaccination rates.

## Methods

The following 9 largest US cities (with surrounding counties except for Chicago, Illinois), representing 40.8 million people, reported neighborhood-level vaccination rates from the beginning of vaccinations through April 13, 2021: New York, New York; Los Angeles, California; Chicago; Houston, Texas; Phoenix, Arizona; Philadelphia, Pennsylvania; San Antonio, Texas; San Diego, California; and Dallas, Texas. In this cross-sectional study, we obtained data on COVID-19 vaccination and death rates for these cites from health authority websites and sociodemographic information from the American Community Survey.^3^ Race and ethnicity were self-identified by the American Community Survey respondents. This study used only deidentified, publicly available data and was therefore exempt from institutional review board approval and informed consent in accordance with the Common Rule and Columbia University policy. We followed the Strengthening the Reporting of Observational Studies in Epidemiology (STROBE) reporting guideline for cross-sectional studies.

We defined neighborhoods using zip codes. In Los Angeles we used communities, a similar level of aggregation. Within each city, we divided neighborhoods into quartiles according to vaccination rate (adults with at least 1 dose) and calculated the mean sociodemographic characteristics and COVID-19 death rates in each quartile. To measure vaccination rates relative to historical disease burden (defined as the cumulative death rate from COVID-19), we tested the association between COVID-19 death rates and vaccination rates within cities using linear regression and conducted a concentration analysis assessing the share of vaccinations administered in neighborhoods with the highest death rates.^4^

We considered *P* < .05 from 2-sided tests to be statistically significant. Analyses were performed using Stata/MP, version 16.0 (StataCorp, LLC).

## Results

We analyzed 1127 neighborhoods with a mean (SD) COVID-19 vaccination rate of 42.3% (13.4 percentage points). Neighborhoods in the lowest quartile had less than half the vaccination rate of those in the highest quartile (27.6% vs 59.7%; Table). Neighborhoods with high vaccination rates had a greater share of White and Asian people and a lower share of Black and Hispanic or Latino people. These neighborhoods also had higher mean incomes, lower poverty rates, and higher 4-year college completion rates. Employment in health care differed little across quartiles, but in neighborhoods with high vaccination rates, these workers were more likely to be health care practitioners or technologists and less likely to be in support occupations.

**Table.** Neighborhood Characteristics by Quartile of COVID-19 Vaccination Rate

Neighborhood characteristics^a^	Quartile^b^				*P* value^c^
	First	Second	Third	Fourth	
Percentage of adults who received at least 1 vaccination dose^d^	27.6	37.1	45.3	59.7	<.001
Race^e^					
Asian	4.1	8.1	12.0	15.2	<.001
Black or African American	24.6	16.2	11.8	6.0	<.001
Indigenous^f^	1.0	1.0	1.1	0.7	.690
White	52.2	56.4	61.9	69.7	<.001
Ethnicity^e^					
Hispanic or Latino	47.2	44.8	31.6	18.1	<.001
Educational level < bachelor’s degree^g^	82.2	74.5	60.8	41.0	<.001
Age 65 y or older	11.4	12.7	14.1	17.3	<.001
Occupation^h^					
Education	4.6	5.3	6.1	6.4	<.001
Health care	9.1	8.9	8.9	9.5	.090
Practitioner	3.9	4.6	5.9	7.6	<.001
Support staff	5.2	4.2	3.1	1.9	<.001
Protective services	2.9	2.4	2.2	1.6	<.001
Income < federal poverty line^e^	22.1	17.2	11.9	8.5	<.001
Median household income, $^i^	51 940	59 065	77 270	107 714	<.001
COVID-19 deaths per 100 000 population^j^	211.6	228.0	198.8	142.8	<.001
Neighborhoods, No.	284	281	284	278	

^a^ Neighborhoods are defined as zip codes, except for Los Angeles, California, which uses a community concept at essentially the same level of aggregation (there are 329 communities vs 300 zip codes).

^b^ Unless otherwise specified, each cell in this table reports a mean percentage for neighborhoods in the given COVID-19 vaccination rate quartile. Quartiles were defined city by city; each column aggregates neighborhoods in the given quartile in all 9 cities.

^c^ *P* values are for tests of the null hypothesis that the means are the same for all 4 quartiles.

^d^ Percentage among people aged 18 years and older. Three neighborhoods with vaccination rates of more than 100% were omitted because of likely data errors.

^e^ Percentage of the total population.

^f^ Indigenous is defined as American Indian, Alaska Native, Native Hawaiian, and other Pacific Islander.

^g^ Percentage of people with less than a bachelor’s degree among people aged 25 years and older.

^h^ Percentage of employed people aged 16 years and older.

^i^ Mean of household income for neighborhoods in the given quartile, where each neighborhood’s household income is taken at its median.

^j^ Excludes data from Phoenix, Arizona, and Dallas, Texas, which did not report deaths by neighborhood.

Historical COVID-19 death rates (from the first COVID-19 deaths through April 13, 2021) were lowest in neighborhoods with the highest vaccination rates, even though these neighborhoods had more older adults. A 10-percentage-point increase in the vaccination rate was associated with 25 fewer historical COVID-19 deaths per 100 000 population (*P* < .001, Figure). Of the 863 neighborhoods with death data, the 209 with the highest death rates accounted for half of all historical COVID-19 deaths but 26% of all vaccinations.

**Figure.**
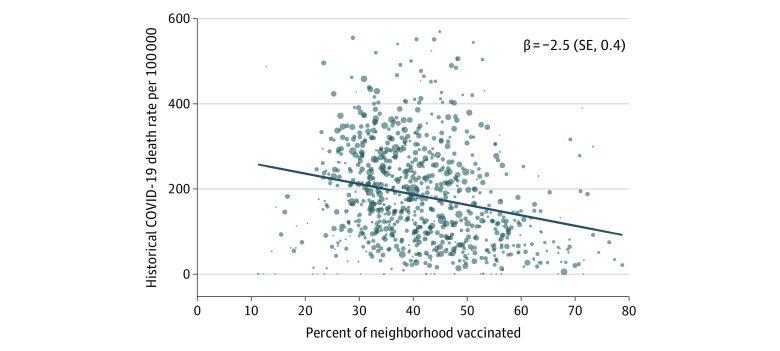
Association Between Neighborhood Historical COVID-19 Death Rate and Vaccination Rate The COVID-19 death rate is per 100 000 total population, and the vaccination rate is the proportion of adults aged 18 years or older who received at least 1 COVID-19 vaccination dose. Each circle represents 1 neighborhood and is scaled according to its total population. Neighborhoods are defined as zip codes, except for Los Angeles, California, which uses a community concept at essentially the same level of aggregation (329 communities vs 300 zip codes). One neighborhood with a vaccination rate of more than 100% was omitted because of a likely data error. The 18 neighborhoods above the 98th percentile of death rates (579 per 100 000) and the 11 neighborhoods with vaccination rates lower than 10% or higher than 80% were trimmed from the plot but were included in the calculation of the line of best fit. The line of best fit controls for city and is weighted by total population. β is the slope coefficient with robust SE reported in parentheses. Data for Phoenix, Arizona, and Dallas, Texas, were excluded from the plot because these cities did not report deaths by neighborhood.

## Discussion

In the 9 largest US cities, COVID-19 vaccination rates were disproportionately high in communities with lower burdens of this disease. This study builds on reports of inequitable COVID-19 vaccination rates by race and ethnicity that were frequently based on incomplete demographic data from states^2^ as well as county-level analyses of early vaccination efforts that found comparatively small differences in vaccination rates by county social vulnerability index.^5^ Using more granular neighborhood-level data, we documented substantial inequities in vaccination rates in the first 5 months of vaccine distribution.

Limitations of this study include potential inaccuracies in vaccination and death reporting, the inability to distinguish the role of supply- and demand-side factors, and limits on the generalizability of the results beyond the included cities. We were also unable to assess the role of vaccine supply and eligibility policies in creating or mitigating disparities. However, given that all US adults have been eligible to receive the vaccine since April 2021 and there is current excess supply, other policies are now more actionable targets to improve vaccination equity.

Inequities in vaccination rates across neighborhoods likely reflect several root causes, including systematic underinvestment in public health in segregated communities, unequal access to health care information and services, and medical racism that drives legitimate mistrust among members of marginalized groups.^6^ The findings of the present study emphasize the opportunity and need for cities to address vaccination inequities in marginalized communities.
